# Prevalence of glucose-6-phosphate dehydrogenase deficiency and sickle cell trait among blood donors in Riyadh

**DOI:** 10.4103/0973-6247.59389

**Published:** 2010-01

**Authors:** Mohammed K. Alabdulaali, Khaled M. Alayed, Abdulaziz F. Alshaikh, Shihab A. Almashhadani

**Affiliations:** *Department of Pathology and Laboratory Medicine, King Khalid University Hospital, Riyadh, Saudi Arabia*; 1 *Department of Haematology Unit, King Khalid University Hospital, Riyadh, Saudi Arabia*; 2 *Department of Blood Bank, King Khalid University Hospital, Riyadh, Saudi Arabia*

**Keywords:** Blood transfusion, glucose-6-phosphate dehydrogenase deficiency, sickle cell trait

## Abstract

**Background and Aims:**

Blood donation from glucose-6-phosphate dehydrogenase (G6PD)-deficient and sickle cell trait (SCT) donors might alter the quality of the donated blood during processing, storage or in the recipient's circulatory system. The aim of this study was to determine the prevalence of G6PD deficiency and SCT among blood donors coming to King Khalid University Hospital (KKUH) in Riyadh. It was also reviewed the benefits and risks of transfusing blood from these blood donors.

**Materials and Methods:**

This cross-sectional study was conducted on 1150 blood samples obtained from blood donors that presented to KKUH blood bank during the period April 2006 to May 2006. All samples were tested for Hb-S by solubility test, alkaline gel electrophoresis; and for G6PD deficiency, by fluorescent spot test.

**Results:**

Out of the 1150 donors, 23 (2%) were diagnosed for SCT, 9 (0.78%) for G6PD deficiency and 4 (0.35%) for both conditions. Our prevalence of SCT and G6PD deficiency is higher than that of the general population of Riyadh.

**Conclusion:**

We recommend to screen all units for G6PD deficiency and sickle cell trait and to defer donations from donors with either of these conditions, unless if needed for special blood group compatibility, platelet apheresis or if these are likely to affect the blood bank inventory. If such blood is to be used, special precautions need to be undertaken to avoid complications in high-risk recipients.

## Introduction

Intracorpuscular red blood cell (RBC) defects might affect its survival, resistance to various stresses and/or interaction with other cells like leukocytes or endothelial cells. It is of high significance if such red cells are to be donated and transfused to a recipient encountering a stressful event. Glucose-6-phosphate dehydrogenase (G6PD) deficiency is an X-linked enzymopathy characterized by inability of RBCs to counterbalance oxidative stress, exposing them to intravascular hemolysis. Hemoglobin-S (Hb-S) is the most common hemoglobin variant, which is brought by an autosomal structural single-point mutation and characterized by poor solubility in the deoxygenated state followed by polymerization leading to RBC shape distortion, rigidity and extravascular hemolysis.

Among all inherited RBC disorders, G6PD deficiency and sickle cell trait (SCT) share the following features: Being highly frequent in many geographical areas and ethnic groups; usually asymptomatic and in stable conditions not altering hemoglobin level, RBC count and indices, hence easily missed by routine complete blood count (CBC) and clinical history taken from a person who has not been screened for them or experienced any acute hemolytic attack. For these reasons, it is not uncommon to encounter persons who are affected by the same conditions as prospective blood donors. There are controversies regarding the quality of blood donated by these two groups during processing, storage or in the recipient's circulatory system.

In the Kingdom of Saudi Arabia (KSA), we have a high frequency of G6PD deficiency and SCT.[1–4] Blood donors are not routinely screened for these conditions in most KSA blood banks and their detection is relied on the pre-donation data. Considering that the majority of our donors are males and the potential alteration in the quality of the donated blood, the present study was conducted to determine the prevalence of G6PD deficiency and SCT among blood donors presenting to King Khalid University Hospital (KKUH) in Riyadh and to review the benefits and risks of utilizing blood from these blood donors.

## Materials and Methods

This cross-sectional study was conducted on 1150 blood samples obtained from blood donors that presented to KKUH blood bank during the period April 2006 to May 2006. They were aged 18 to 50 years. Blood samples were collected in ethylene diamine tetraacetic acid (EDTA) tubes and then kept at 2-6°C and processed within 24 hours after collection. All samples were first tested by automated analyzer for CBC; then blood smears were prepared, followed by investigations to detect Hb-S by solubility test, alkaline gel electrophoresis; and G6PD deficiency, by fluorescent spot test.

The above-mentioned 1150 donors represented all the donors that presented during the period of this study and who answered the pre-donation questionnaire for selection, which did not include any direct questions regarding SCT or G6PD deficiency, and fulfilled the inclusion criteria.

## Results

There were 1137 (98.87%) male donors and 13 (1.13%) female donors. Majority (1058, 92%) of blood donors were Saudis. Out of the 1150 donors, 23 (2%) were diagnosed for SCT, 9 (0.78%) for G6PD deficiency and 4 (0.35%) for both conditions. All were males and Saudis. Their Hb levels were normal [123-156 g/L (mean, 143 g/L); and their RBC count, 3.88-5.47 × 10^12^/L (mean, 5.1 × 10^12^/L); MCV, 68.9-92.4 fl (mean, 82.8 fl); MCH, 23.4-31.6 pg (mean, 28.4 pg); and RDW, 12.4-14.5 (mean, 13.3) were normal in all cases, except 1 case, which showed microcytic hypochromic red cells with normal RDW and target cells that raised the possibility of a coexistent alpha-thalassemia trait.

## Discussion

In KSA we have a high frequency of G6PD deficiency,[1] with regional variability; the ranges are 0-0.398 (mean, 0.0905) in males and 0-0.214 (mean, 0.041) in females[5] with the highest frequency being in eastern areas.[6] SCT also is a highly frequent disorder in KSA, with a range of 0-0.2588 (mean, 0.0736),[2–4] with the highest frequency being in western, southern and eastern areas.[2,3,6,7] In Riyadh, the capital city of KSA, located in its central region, the frequency of SCT is 0.01.[2]

Our results showed a higher prevalence of SCT and G6PD deficiency compared to that in the general population of Riyadh. The inter-regional movement of population toward the central and big cities can explain that; for this reason, the adult population of Riyadh might include persons from other regions, including those moving from areas with high prevalence of G6PD deficiency and SCT. Majority of the donors in this study were males, and this also will contribute in increasing the prevalence of G6PD deficiency in the studied population.

The use of G6PD-deficient blood has been studied for simple and exchange transfusions. It has been proposed that several biochemical changes and depletion in the antioxidant defense system occur on storage of G6PD-deficient blood.[8,9] In infants, Mimouni *et al.*, described hemolysis in 2 preterm infants who received G6PD-deficient donor blood. Neither infant received drugs known to be hemolytic. Both infants required further intervention by exchange transfusions.[10] In the exchange transfusion practice with G6PD-deficient blood, a lesser drop in post-exchange total serum bilirubin, prolongation of the duration of phototherapy, the need for repeat exchange transfusions and occurrence of massive intravascular hemolysis in infants have also been reported.[11–13] The above complications have been reported in patients with normal G6PD activity. However, in high-prevalence areas of G6PD deficiency, more than 40%[14] of neonates with jaundice that might require exchange transfusion are G6PD-deficient, hence the incidence and severity of these complications are expected to be higher.

The use of G6PD-deficient blood in adults did not show significant adverse clinical consequences following transfusion except biochemical changes reflecting mild hemolysis in one of the prospective studies.[15–17] This can be due to higher blood volume of the patient and the effect of the normal blood transfused along with the G6PD-deficient one if the patient received 2 units or more.[15,18] In Mediterranean and Middle East, the interaction between G6PD Mediterranean mutation and alpha-thalassemia can ameliorate the clinical complications.[19]

SCT has been considered as a benign condition. However, life-threatening complications may occur, and these clinical complications have been reported by several *in vitro* studies in which Hb-AS red cells showed abnormality of their filterability.[20] RBCs collected from SCT donors frequently occlude WBCs' reduction filters. The main cause of this filtration failure is hemoglobin polymerization.[21–24] There are many parameters that might further affect the adequacy of WBC filtration, including temperature, platelets, osmolarity, type of anticoagulant, time of storage and oxygen saturation of the blood unit.[21,24,25] Failure of adequate WBC reduction has been shown to increase the incidence of febrile non-hemolytic transfusion reaction, transmission of leukocyte-associated viruses and HLA alloimmunization.[23] Storage of Hb-AS whole blood in large-capacity oxygen-permeable bags increases oxygen tension and allows more effective WBC reduction by filtration.[22,24]

The acute congestive crisis-like syndrome associated with Hb-AS red cell exchange transfusion is rarely seen, mainly due to the modifying effect of a concurrent normal blood transfusion.[18]

## Conclusion

Transfusion with G6PD-deficient blood carries a potential risk of hemolytic complications, especially if it is used for exchanged blood transfusion in neonates. On the other hand, the blood donated from SCT donors, apart from its undesired effects if transfused to sickle cell disease patients, also leads to WBC filtration failure. For these reasons, in high-prevalence areas for SCT and/or G6PD deficiency, we recommend to screen all units for these conditions and to defer donors with these conditions, unless if needed for special blood group compatibility, platelet apheresis or if it is likely to affect the blood bank inventory. For the latter reason, donation can be accepted but G6PD-deficient blood should be labeled and should not be released to transfuse a G6PD-deficient patient or to exchange in the pediatric age group, particularly neonates. On the other hand, SCT units need to be labeled too, and such blood is better stored in bags that allow increased O_2_ saturation and not used to transfuse sickle cell disease patients or in patients where WBC filtration is of great concern. In blood banks with limited resources where screening for SCT and G6PD deficiency is not feasible, we advise to screen the units that are likely to be transfused to high-risk recipients, particularly if single-unit transfusion is going to be undertaken.
